# Recent Advances in Investigation, Prevention, and Management of Healthcare-Associated Infections (HAIs): Resistant Multidrug Strain Colonization and Its Risk Factors in an Intensive Care Unit of a University Hospital

**DOI:** 10.1155/2019/2510875

**Published:** 2019-06-20

**Authors:** Dana Carmen Zaha, Rita Kiss, Csaba Hegedűs, Rudolf Gesztelyi, Mariann Bombicz, Mariana Muresan, Annamaria Pallag, Miklos Zrinyi, Denes Pall, Cosmin Mihai Vesa, Otilia Micle

**Affiliations:** ^1^Department of Preclinical Disciplines, University of Oradea, Faculty of Medicine and Pharmacy, 1^st^ December Square 10, Oradea, 410068, Romania; ^2^Department of Pharmacology and Pharmacotherapy, Faculty of Medicine, University of Debrecen, Nagyerdei krt. 98, H-4032 Debrecen, Hungary; ^3^Department of Pharmacy, University of Oradea, Faculty of Medicine and Pharmacy, 1st December Square 10, Oradea, 410068, Romania; ^4^Coordination Center for Drug Development, Faculty of Medicine, University of Debrecen, Nagyerdei krt. 98, H-4032 Debrecen, Hungary

## Abstract

Active screening for resistant multidrug strain carriers remains an important component of infection control policy in any healthcare setting indifferent of financial and logistical costs. The objective of our study was to determine the spectrum of bacterial colonization individually among intensive care unit patients. A retrospective observational study was performed in the Intensive Care Unit of Emergency Clinical County Hospital of Oradea during 2017. Medical records of the patients were used for evaluation of source of ICU admission, previous antibiotic therapy, comorbidities, and length of hospital stay. Nasal and groin swabs for MRSA detection and rectal swabs for ESBL, VRE, and CRE detection were collected upon ICU admission of all patients in the first 24 hours and after 7 days. Swab samples were processed for isolation and identification of these resistant multidrug strains. Bacterial colonization on admission was detected in a quarter of patients included in the study. Carbapenemase-producing bacteria were the most common colonizers (21.16%). On admission, 12.06% of patients have been colonized by ESBL-producing members of the family Enterobacterales. Risk factors for colonization on admission to the ICU were chronic liver diseases and chronic renal failure for ESBL infection and chronic liver disease for CRE in male patients. Evaluation of Carmeli's score for male patients showed association only with CRE colonization. Chronic renal failure was found as risk factor for ESBL colonization in female patients. The prevalence of MRSA was 5.23% and less than 1% for VRE. There was no association between any risk factors studied and the presence of* S. aureus* or VRE upon admission. The 7-day ICU stay also proved to be an increased risk for ESBL and CRE infection.

## 1. Introduction

Infections associated with medical care (nosocomial) represent a worldwide problem despite the advancement in therapeutic technologies [1]. Intensive care unit (ICU) patients are more affected by these infections caused by special pathogens contributing to prolonged intensive care unit stays, increased morbidity and mortality, and increased resource utilization. Special interest is directed at the study of these special microorganisms resistant to multiple antimicrobials, which are growing at higher rate in the ICU setting, leading to higher treatment costs, morbidity, and mortality [2–6]. An important risk factor for nosocomial infection is prior colonization [7, 8]. Other clinical reports show different data about the incidence and patterns of colonization of multidrug-resistant bacteria [9–11].

As a definition, multiple drug resistance (MDR) is nonsusceptibility of a strain to at least one agent in three or more antibiotic classes [12]. In ICUs, the identified multidrug-resistant microorganisms include both Gram-positive bacteria (*Staphylococcus aureus* and* Enterococcus *spp.) and Gram-negative bacilli (*Escherichia coli*,* Acinetobacter baumannii*,* Pseudomonas aeruginosa*,* Proteus *spp., and* Klebsiella *spp.). Methicillin-resistant* Staphylococcus aureus* (MRSA), vancomycin-resistant* Enterococcus* spp. (VRE), extended-spectrum beta-lactamase producers (ESBL), carbapenemase-resistant Enterobacteriaceae (CRE), and multidrug-resistant Gram-negative bacteria (MDR) are well described [13–16]. MRSA could be a major cause of severe nosocomial infections (bloodstream infections, urinary tract infections, and pneumonia) contributing to the high mortality because of its resistance to a variety of antibiotics [17]. Vancomycin-resistant* Enterococcus* (VRE) has the same difficulties in treating infections or colonization [18]. Gram-negative organisms are important causes of the same nosocomial infections [19, 20]. ESBL Gram-negative producers are frequently isolated especially in ICU patients with multiple comorbidities and they are associated with high morbidity and mortality, due to the limited therapeutic options [21]. The narrowing of treatment strategies and the limited availability of future underdevelopment drugs lead to spreading of MDR-GN bacterial infections [22, 23]. In recent years, Food and Drug Administration (FDA) has approved the usage of new antibiotic combinations such as ceftolozane/tazobactam and ceftazidime/avibactam that are proven to be effective against MDR-GN, but the emergence of resistant strains is just a matter of time [16, 24]. Antibiotic resistance was discovered with the discovery of antibiotics, and a significant number of the analysed strains isolated from food products showed resistance to different antibiotic classes and the molecular techniques revealed that many of these possess either one or multiple genes that confer resistance to different antibiotics, like presence of ESBL and AmpC production [19, 25].

Bacterial colonization of the skin and mucous membranes of patients with these microorganisms usually precedes infections. Colonizers are part of the commensal bacterial flora of the skin that protects it from colonization with pathogenic bacteria; therefore they do not cause any problems usually. These different colonies may disappear spontaneously or be cleared under the action of disinfectant and antimicrobial agents. However, they can also turn into pathogenic bacteria if the activity of the skin surface antimicrobial proteins (AMPs) is compromised or the strains acquire new genes that modify the disease-producing capacity of the bacteria [26, 27]. When the protective barriers of skin and mucous membranes are compromised or immunity deficiency develops, these organisms overwhelm the body, resulting in an infection [28]. Screening of ICU patients upon admission for colonization by MDR strains, handling them with caution and isolation of colonized patients, could prevent nosocomial infections [29]. The purpose of this screening is to prevent spreading of these organisms to other ICU/non-ICU patients, especially those vulnerable to drastic consequences in case of transmission. A study reported that the screening of ICU patients upon admittance for the presence of MRSA and VRE despite special measures applied to isolate carriers did not result in a reduction of ICU-acquired infection with the aforementioned pathogens [29]. Instead, a recent review of literature published by Glick et al. implies that screening to universal MRSA carriage upon admission may reduce the risk of MRSA infections but the power of evidence was weak to support screening programs [30].

There are several scoring system models in use to estimate the severity of critical illness and to predict mortality among ICU patients. The acute physiology and chronic health evaluation (APACHE) model was first introduced, followed by APACHE II and APACHE III including data from American hospitals [31]. Another model, the simplified acute physiology score (SAPS), was instituted in 1985, followed by the revised editions SAPS II and SAPS III as an alternative to the APACHE score. SAPS I is employing 14 of the original 34 parameters used in the APACHE system and SAPS II and SAPS III included data of 12 parameters from the first 24 hours in the ICU [32–34].

In patients who have severe comorbidities and/or are immunocompromised, the multiple hospitalizations and the long-term antibiotic therapy sometimes in combination represent a high risk in the selection and the colonization and infection with resistant or multidrug-resistant strains [35]. The expected chance with which the bacteria might be resistant to the antimicrobial treatment can be assessed using Carmeli's score [36–38].

This study was undertaken to evaluate the prevalence and the spectrum of bacterial colonization in patients admitted to the ICU of the Emergency Clinical County Hospital of Oradea, and to assess the predisposing risk factors for colonization.

## 2. Materials and Methods

### 2.1. Data Collection

The study was conducted at Emergency Clinical County Hospital of Oradea, with a 45-bed ICU that receives patients from within the hospital as well as ones referred from outside. It manages approximately 5600 critically ill patients annually. After ethical clearance by the institution and after receiving informed consent from the patient or from the next of kin (in case of unconscious patient), an active surveillance of 1971 adult admitted patients was done between January and December 2017.

Demographic characteristics, source of ICU admission, previous hospitalization, previous antibiotic therapy, comorbidities, length of hospital stay, and outcome were recorded and analysed. In order to identify the risk factors for colonization with multidrug-resistant strains, the diagnostics have been grouped and analysed as follows: respiratory (pneumonia, bronchial asthma, and chronic obstructive pulmonary disease) and cardiovascular diseases (hypertension, myocardial infarction, chronic heart failure, coronary heart disease, chronic atrial fibrillation, and stroke), chronic renal failure and diabetic nephropathy, chronic liver diseases (chronic hepatitis, hepatic cirrhosis, hepatic encephalopathy, and chronic hepatic insufficiency), cancer, hematologic malignancy (lymphoma and leukaemia), immunosuppressive status (chronic obstructive pulmonary disease, organ transplant, chronic autoimmune diseases, and AIDS), and diabetes mellitus.

Medical history of the patients, results of physical examination, and multiple laboratory parameters were analysed to calculate Carmeli's score in order to identify patients susceptible to be colonized with multidrug-resistant bacteria at the beginning of the hospitalization. The scoring and stratification were based upon the presence of Carmeli's risk factors [36, 37]. Risk factors were ranked with 1, 2, or 3 points according to the prediction for infection with susceptible, resistant, or multidrug-resistant microorganisms. Patients were scored as 1 to 3 according to the severity of the following factors: the degree of contact with the healthcare system and invasive procedures; antibiotic treatment; and patient characteristics such as age, various comorbidities, or immunosuppressed status. The highest numeric value of the three criteria represents final value of Carmeli's score (1, 2, or 3). The final score allowed us to classify patients as follows: score 1 (community-acquired infections with microorganisms susceptible to classic antibiotics), score 2 (probably healthcare-associated or community-acquired infections but with high probability of resistant or multidrug-resistant strains), and score 3 (maximum prediction for nosocomial infections with resistant or multidrug-resistant strains) [38].

### 2.2. Microbiological Procedures

Nasal, groin, and/or rectal swab samples were collected to assess bacterial colonization during the first 24 hours of ICU admission from all study participants. The same samples were collected 7 days after admission for patients who remained hospitalized for that time period. Nasal and respective groin swab samples were collected by wiping the swab in circular motion while simultaneously rotating it in 360° and exerting gentle pressure on the surface. Rectal samples were collected by inserting the swab 1 cm into the rectum while rotating the swab in 360°. Swabs collected were placed in sterile round bottom tubes containing 1 ml sterile Amies transport medium and immediately transferred to the laboratory for analysis. Collected nasal and groin samples were screened for MRSA, while rectal swabs were screened for VRE, ESBL, and/or CRE. The strains have been considered multidrug-resistant if they displayed resistance to more than three antibiotic classes. Patients were considered to be colonized by any of the organisms studied if one or more of those organisms was/were isolated from one or more of the swabbed sites.

#### 2.2.1. MRSA

Nasal and groin samples collected were cultured on MRSA CHROMagar (CHROMID® MRSA, bioMérieux) and incubated at 37°C for 24 hours. Plates were examined for green MRSA colonies; other colours were disregarded. In addition, the latex agglutination test was performed to identify penicillin-binding protein, PBP2A. The isolates were confirmed as MRSA by disc diffusion test using 30 *μ*g cefoxitin disc on Mueller-Hinton agar according to European Committee on Antimicrobial Susceptibility Testing (EUCAST) guidelines. Quality control was done by using* Staphylococcus aureus *ATCC 25923.

#### 2.2.2. VRE

Rectal swab samples were cultured on selective chromogenic medium (CHROMID® VRE) for direct identification of* E. faecalis* and* E. faecium*. The medium allowed the detection of acquired vancomycin-resistant strains (vanA and vanB) by selective coloration of the colonies as follows: blue-green for* E. faecalis *and violet for* E. faecium*. For quality control* E. faecalis *ATCC 51299 was used.

#### 2.2.3. ESBL

All the rectal swabs were processed for isolation of ESBL-producing GN bacteria. Gram-negative bacteria were isolated on ESBL chrome agar (CHROMID® ESBL, bioMérieux). After incubation for 24 hours, it is easy to read results based on the specific coloration.* Escherichia coli *shows a pink to burgundy coloration (ß-glucuronidase-producing colonies),* Klebsiella*,* Enterobacter*,* Serratia*, and* Citrobacter *display a green/blue to brownish green coloration (ß-glucosidase-producing colonies),* Proteus*,* Providencia*, and* Morganella *appear in dark to light brown colonies (deaminase-expressing strains), and* Acinetobacter *spp. shows a cream coloration. ESBL producers were confirmed phenotypically by the double-disk synergy test using clavulanic acid and third-generation cephalosporins. Disks of third-generation cephalosporins and amoxicillin-clavulanic acid were kept 15–20 mm apart, centre to centre, on inoculated Mueller-Hinton agar. The plates were incubated at 35°C–37°C for 18–24 hours. A clear extension of the edge of the inhibition zone of any of the third-generation cephalosporins towards the amoxicillin-clavulanic acid disk was interpreted as positive for ESBL production.* Escherichia coli *ATCC 25922 was used for quality control.

#### 2.2.4. CRE

The rectal swabs were analysed as well for the presence of GN bacteria with a reduced susceptibility to most of the carbapenem agents. We used selective chromogenic medium for the screening of carbapenemase-producing Enterobacteriaceae (CHROMID® CARBA).* Escherichia coli *displays a pink to burgundy coloration,* Klebsiella *shows green coloration, and* Acinetobacter *spp. appears in colorless or cream colonies. Any carbapenem-resistant isolate was investigated for phenotypic carbapenemase production using the carbapenem inhibition method [39]. For quality control, Klebsiella pneumoniae ATCC 25955 was used.

All isolates were identified to the species level by using MALDI-TOF mass spectrometry. The antibiotic susceptibility pattern was obtained by the Kirby–Bauer disc diffusion method on Mueller–Hinton agar and Vitek-2 according to the EUCAST guidelines.

Patients were considered colonized by any of the organisms studied if one or more of those organisms were isolated from at least one of the swabbed sites. ICU-acquired infection was defined if any of the studied pathogen colonies were detected 7 days after ICU admission in previously negative patients.

### 2.3. Data Analysis

Data were described as mean ± standard deviation (SD), number of cases, or percentages where appropriate. For the statistical analysis of the contingency tables, Chi-square test and Fisher's exact test were used. Statistical significance was considered when p<0.05. All statistical calculations were done with GraphPad Prism 7.00.

## 3. Results

### 3.1. Demographic Data of Patients

The demographic characteristics and the possible risk factors for infection are summarized in Table 1. A total of 1971 patients, 1107 males and 864 females, were included in this study. The male : female ratio was 1.28 : 1 (56.16% male and 43.83% female). Mean age range was 65.13±14.65 years for males and 70.15±14.13 for females. More than half of the patients were above 65 years of age. One-third of patients (31.6%) had been hospitalized in the previous months, 1473 of 1971 (74.73 %) have been treated with antibiotics, and 44 (2.23%) patients with chronic renal failure came in for regular dialysis.

Carmeli's score was an average of 1.54±0.66 in the male and 1.75±0.77 in female patients. The interpretation of our results shows that the patients included in the study are mostly with probably healthcare-associated or community-acquired infections but with high probability of resistant or multidrug-resistant strains. Among our patients, 96 (4.87%) were surgical patients, from whom 23 (23.95%) received a perioperative infection prophylaxis; therefore the rest of the patients were medical patients and the majority had received at least one group of antibiotics. The main three chronic disease groups presented by patients included in the study were cardiovascular disease, diabetes mellitus, and respiratory diseases regardless of the gender.

### 3.2. Bacterial Pattern of Colonization upon Admission

Overall, 1971 patients were sampled at one or more sites (nasal, groin, and rectal region) to assess colonization. Bacterial colonization (of 1 or more sites) upon admission was detected in 494 patients (25.06%). Evaluation of spectrum of bacterial colonization on admission shows that there are no significant differences between male and female patients. The results of the cultures from different swabbed sites obtained from the male and female study participants at admission are presented in Table 2.

A number of 687 groin and nasal swabs were collected to detect MRSA; overall positivity has been 5.24%, 4.72% in male and 5.88% in female participants. If we compare the positivity rates between the two sites, groin and nasal cavity, they were 2.91% for the groin and 3.35 % for the nasal. In the same Gram-positive microorganisms, our results show a low rate of VRE colonization, less than 1%, slightly higher in female patients (0.25% versus 0.59%). 162 from 1343 (12.06%) of the studied patients were colonized upon admission with one or more of ESBL-producing organisms. Among the colonizing organisms, 50.76% of the ESBL-producing organisms belonged to the* E. coli *species. There were no significant differences between males (11.96%) and females (12.19%). Among Gram-negative bacteria with a reduced susceptibility to most carbapenem agents (CRE), Enterobacterales were the most prevalent (21.16%). In this order, the principal colonizer organism was* E. coli *(68.07%), followed by* Enterobacter *spp. (11.5%),* Proteus *spp. (7.04%), and* Klebsiella *spp. (4.2%). Nonfermenters were identified as* Pseudomonas aeruginosa *and* Acinetobacter baumannii *and they are almost in the same proportion in males and females, being less than 1% (11 from 1389).

### 3.3. Bacterial Pattern of Colonization at 7 Days

Patients who were hospitalized for more than 7 days were retested using the same nasal, groin, and rectal swabs for MRSA, VRE, ESBL, and CRE infection (see Table 3).

According to our results, we found that more than 90% of patients were admitted to the ICU without MRSA or VRE infection regardless of gender. Approximately 3 to 4% of patients acquired MRSA infection during the ICU stay, and in male participants 2.99% were found with VRE positivity. We tested the patients during the first day of admission to the ICU and 4% of male and 7.69% of female patients were found to be positive to MRSA upon admittance, and all of them were found to be cleared of infection during the first week of stay. Regarding ESBL and CRE, we found that approximately 88% arrived to the ICU without being colonized, but during their first 7 days of hospitalization nearly 40% got infected with ESBL, while regarding CRE this number is closer to 33%. Upon admittance, 10-15% of patients were positive either to CRE and/or ESBL, and only half of them were found to be clear of infection during their first week of hospitalization.

### 3.4. Risk Factors for Colonization

According to our analysis, there was no significant association between age, Carmeli's score, and any of the examined comorbidities regarding MRSA or VRE infection upon admittance neither in male nor in female patients. In male patients chronic renal failure and liver disease and in female patients only chronic renal failure proved to be significant risk factors for acquiring ESBL infection, with an approximately 5 times higher risk compared to the rest of the participants. Contrarily, cardiovascular diseases in male patients seem to be posing a lower risk of ESBL infection. Regarding CRE, Carmeli's score and chronic liver diseases in male patients, and chronic renal failure in female patients represents a higher risk of infection, while cardiovascular diseases in female participants show a lower risk of colonization (see Tables 4 and 5).

The risk assessment of infection at 7 days showed statistical significance for ESBL and CRE in both male and female patients (see Table 6).

## 4. Discussion

In this study, we have analysed the incidence and pattern of bacterial colonization including MDR Gram-positive (MRSA, VRE) and Gram-negative (ESBL, CRE) germs in ICU patients upon admission and after 7 days. Multidrug resistance in Enterobacterales and nonfermenting Gram- negative bacilli represent a real challenge to the hospitals or healthcare institutions. Carriage or infections with these isolates can result in compromised treatment options and high mortality rates in patients. Therefore, early detection of infected or colonized patients is compulsory for the best patient management and to prevent the patient-to-patient transmission or environmental contamination. In addition, identifying the risk factors associated with MDR bacterial infections and the most commonly occurring MDR strains may be of use to hospital antimicrobial drug management and the prevention of nosocomial contamination [40]. Most investigations of risk factors for multidrug-resistant strains have been hospital-based and focused on them. The risk factors can be exposure to antibiotics and multidrug-resistant strains in the environment (recent antibiotics and/or hospitalization, history of multidrug-resistant strains, colonization pressure, comorbidity, and dialysis) and special conditions that facilitate colonization and infection (illness severity, wounds, indwelling devices, etc.). Some risk factors have more importance than others, but they can be evaluated and updated. The prevalence and patterns of resistance vary significantly by geographical area, location, size, and facility type, with each institution having a unique pattern. Some hospitals screen all admissions for multidrug-resistant strains colonization. Moreover, an important recommendation is to follow the guidelines regarding the prevention of nosocomial infections. Our institution recently introduced Carmeli's score for evaluation of risk factors for infections with resistant or multidrug-resistant bacteria and it allows doing a prediction. A better diagnostic information includes culture of the causative pathogen and the screening for them was performed only for one year (2017). Rising rates of Gram-negative multidrug-resistant bacteria have been documented. Therefore, it is essential to collect these data and not to assume that our ICU issues are the same as those reported by others.

The overall MRSA carriage rate of 5.2% found in our study is less than 22.5%, which was reported by Ray et al. [41], and greater than 3%, as reported by other scientists [42, 43]. The slightly higher prevalence of MRSA carriage found in the present study could be explained by the relatively small number of patients examined, and the significantly higher rate of infection in Ray's report could be due to the multiple sites of sampling which could detect the presence of MRSA with more accuracy. One of the most prevalent sites of MRSA has been reported to be the nose and our data is in agreement with that observation [41]. The incidence of MRSA infection in the ICU was nearly the same as that on admission in the case of male patients, but females were more likely to be affected on admission than being infected in the hospital. We observed that the rate of MRSA eradication during the hospitalization was 100%, which can be attributed to the absence of correlation of the MRSA carriage with different risk factors and to the correctly chosen and applied antibiotic therapy. Previous studies have identified various risk factors for VRE colonization including advanced age, central venous catheterization, extended hospitalization, haematologic malignancies, haemodialysis, exposure to multiple antibiotics, and prolonged duration of antibiotic therapy [44]. Karki et al. in a study detected 17.5% prevalence of VRE colonization on the day of screening [45]. Contrarily, our results show a significantly low rate of VRE colonization upon admission (0.4%), which is slightly higher in female patients. Seven days of ICU stay proved no higher risk of VRE infection regardless of gender. The beta-lactam antimicrobial agents are among the most commonly used classes of antibiotics. Consequently, resistance to *β*-lactams by production of *β*-lactamases is the most common cause of resistance to these drugs. Majority of the ESBLs are found in* Klebsiella *spp. and* Escherichia coli *[46]. Our study results identified 12.06% prevalence rate for ESBL producers and it correlates well with data from an Iranian report but a Canadian research group found less occurrence in their patient screening study [47, 48]. Regarding ICU stay, we concluded that at least 7 days of admittance poses an approximately five times higher risk of ESBL contamination. According to our results, chronic renal failure and liver diseases might contribute to such high prevalence; however, we also found that more than half of the already ESBL-infected patients staying in the ICU were still carrying the MDR strain after one week of hospital care. As a subclass of beta-lactam antibiotics, the carbapenems are usually applied as spare drugs in the treatment of severe bacterial infections that are resistant to the commonly used antibiotics. The current study showed very high incidence (21.06%) of CRE infection and more in the female than male patients at admission and after 7 days. Upon admittance, more than 10% arrived already carrying the carbapenemase expressing strain, and half of these colonizers were positive to CRE after 7 days. They might have contributed to that during the hospitalization; more than 30% of the CRE-negative patients acquired CRE infection. Apart from hospitalization, chronic liver diseases and Carmeli's score proved to be a statistically significant risk factor in men, while in the case of women we found a tendency that points to the possible connection of chronic renal failure to CRE infection.

According to our current results, we can report that nosocomial infections can occur in the Intensive Care Unit of Emergency Clinical County Hospital of Oradea. The most common isolates were ESBL and CRE strains, but in comparison Gram-positive MDR bacteria such as MRSA and VRE were detected in a significantly less proportion. It is promising that, in addition to the lower level of colonization, MRSA and VRE were showing a better response to antimicrobial therapy compared to GN MDR bacteria. However, ESBL and CRE pose a difficult problem by either medical or economic reasons. The number of nosocomial GN infections was remarkably high, which can be attributed to the fact that more than half of the originally infected patients remain carriers even after being a week after admittance into intensive care, and it should also be noted that the hospital environment contributes to the selection of strains displaying one or more resistance mechanisms against the antibiotics; therefore we have to prepare for the appearance of more persistent bacteria in the future. We must underline the importance of rational antibiotic strategies, the appropriate personal hygiene and preventive methods, and the consideration of auxiliary medical therapies, such as the application of natural compounds that might offer favourable alternatives based on their antioxidant and disinfectant properties [49, 50]. Finally, the risk factor assessment should be done before selecting empiric antibiotic therapy and it could improve the prognosis of some patients and help the development of infection control policies and procedures, as well as the review of antibiotic utilization and its relationship to local antibiotic resistance patterns, together with the development of guidelines for the rational use of antimicrobial therapy.

## 5. Conclusion

Our study is in accordance with other findings and supports the importance of identifying and managing risk factors involved in the mechanism of colonization of the human patients with potentially multidrug-resistant pathogenic bacteria during hospitalization, especially in the intensive care unit.

## Figures and Tables

**Table 1:** Clinical characteristics of the patients (n= 1971).

	Male	Female

Number of patients	1107	864
Mean age (years)	65.13±14.65	70.15±14.13
Age > 65 years	599	618
Carmeli score	1.54±0.66	1.75±0.77
Previous hospitalization	349	274
Antibiotic treatment	825	648
Surgical patients	53	42
Perioperative infection prophylaxis	13	10
Respiratory diseases	173	140
Cardiovascular diseases	516	437
Chronic renal failure	30	14
Chronic liver diseases	47	27
Cancer	96	70
Hematologic malignancy	3	0
Diabetes mellitus	183	210

**Table 2:** Spectrum of bacterial colonization upon admission. MRSA-N: MRSA detected from nasal cavity; MRSA-G: MRSA detected from groin area.

	Male			Female		
	Number of patients [n]	Positivity [n]	Positivity [%]	Number of patients [n]	Positivity [n]	Positivity [%]

MRSA	381	18	4.72%	306	18	5.88%
MRSA-N	381	13	3.41%	306	10	3.26%
MRSA-G	381	9	2.36%	306	11	3.59%
VRE	400	1	0.25%	340	2	0.59%
ESBL	744	89	11.96%	599	73	12.19%
CRE	833	166	19.92%	556	127	22.84%

**Table 3:** Bacterial colonization of male and female patients upon 7 days of admission. NEG-NEG: no infection was found at admittance and at 7 days. NEG-POS: no infection was found at admittance but the patient was found to be infected at 7 days. POS-NEG: infected at admittance but no infection was found at 7 days. POS-POS: infected at admittance and the patient was found to be infected at 7 days.

	Male				Female			
	MRSA	VRE	ESBL	CRE	MRSA	VRE	ESBL	CRE
NEG-NEG	93%	97.01%	52%	63.11%	88.46%	100%	49.66%	54.86%
NEG-POS	3%	2.99%	33.33%	25.41%	3.85%	0%	37.24%	32.64%
POS-NEG	4%	0%	6%	4.92%	7.69%	0%	6.90%	4.86%
POS-POS	0%	0%	8.67%	6.56%	0%	0%	6.21%	7.67%

**Table 4:** Analysis of risk factors related to bacterial infections upon ICU admission in male patients. OR: Odds Ratio; ∗: p<0.05; ∗∗: p<0.01; ∗∗∗: p<0.001.

Risk factor	MRSA colonization				VRE colonization			
	Present [n]	Absent [n]	OR	P value	Present [n]	Absent [n]	OR	P value

Age > 65 years	10	208	0.9315	>0.9999	1	219	Infinity	>0.9999
Carmeli's score	10	161	1.568	0.4675	1	183	Infinity	0.46
Respiratory diseases	5	68	1.669	0.3575	0	67	0	>0.9999
Cardiovascular diseases	8	165	0.96	>0.9999	1	193	Infinity	0.485
Chronic renal failure	0	9	0	>0.9999	0	9	0	>0.9999
Chronic liver diseases	0	21	0	0.6123	0	20	0	>0.9999
Cancer	3	30	2.22	0.1972	0	34	0	>0.9999
Hematologic malignancy	0	1	0	>0.9999	0	2	0	>0.9999
Diabetes mellitus	4	53	1.671	0.3257	0	70	0	>0.9999

Risk factor	ESBL colonization				CRE colonization			
	Present [n]	Absent [n]	OR	P value	Present [n]	Absent [n]	OR	P value

Age > 65 years	53	352	1.267	0.3101	87	312	1.334	0.1137
Carmeli's score	46	273	1.497	0.0866	80	246	1.686	0.0039∗∗
Respiratory diseases	15	109	1.015	>0.9999	29	90	1.406	0.168
Cardiovascular diseases	32	312	0.6172	0.0415∗	57	282	0.7463	0.1289
Chronic renal failure	6	10	4.663	0.0074∗∗	7	14	2.117	0.156
Chronic liver diseases	12	20	4.948	0.0001∗∗∗	12	21	2.474	0.022∗
Cancer	9	47	1.455	0.2914	18	51	1.518	0.1538
Hematologic malignancy	1	1	7.432	0.2251	0	2	0	>0.9999
Diabetes mellitus	12	104	0.8257	0.6422	28	90	1.347	0.2105

**Table 5:** Analysis of risk factors related to bacterial infections upon ICU admission in female patients. OR: Odds Ratio; ∗: p<0.05; ∗∗: p<0.01.

Risk factor	MRSA colonization				VRE colonization			
	Present [n]	Absent [n]	OR	P value	Present [n]	Absent [n]	OR	P value

Age > 65 years	13	207	1.017	>0.9999	2	241	Infinity	>0.9999
Carmeli score	9	166	0.7349	0.6254	2	189	Infinity	0.5062
Respiratory diseases	6	53	2.217	0.128	1	66	4.121	0.3558
Cardiovascular diseases	11	159	1.275	0.8077	1	176	0.9205	>0.9999
Chronic renal failure	0	4	0	>0.9999	0	5	0	>0.9999
Chronic liver diseases	1	7	2.361	0.3879	0	12	0	>0.9999
Cancer	2	24	1.375	0.6571	1	32	9.563	0.185
Hematologic malignancy	-	-	-	-	-	-	-	-
Diabetes mellitus	3	68	0.6471	0.7734	0	82	0	>0.9999

Risk factor	ESBL colonization				CRE colonization			
	Present [n]	Absent [n]	OR	P value	Present [n]	Absent [n]	OR	P value

Age > 65 years	50	376	0.8673	0.5843	83	311	0.8217	0.4234
Carmeli score	44	288	1.254	0.3828	64	239	0.8879	0.6051
Respiratory diseases	9	81	0.7726	0.6011	28	74	1.44	0.1472
Cardiovascular diseases	35	277	0.8279	0.4564	49	238	0.5433	0.0038∗∗
Chronic renal failure	4	6	5.024	0.0241∗	4	4	3.624	0.0754
Chronic liver diseases	3	15	1.46	0.472	6	12	1.809	0.2501
Cancer	8	37	1.627	0.2362	10	38	0.9246	>0.9999
Hematologic malignancy	-	-	-	-	-	-	-	-
Diabetes mellitus	19	118	1.217	0.5518	28	108	0.8921	0.7208

**Table 6:** Analysis of risk factors related to bacterial infections after 7 days. OR: Odds Ratio; ∗: p<0.05; ∗∗∗: p<0.001.

	MRSA colonization				VRE colonization			
	Present [n]	Absent [n]	OR	P value	Present [n]	Absent [n]	OR	P value

Male	3	93	0.6505	0.7801	2	65	11.94	0.0553
Female	4	92	0.6957	0.6163	0	181	0	0.5457

	ESBL colonization				CRE colonization			
	Present [n]	Absent [n]	OR	P value	Present [n]	Absent [n]	OR	P value

Male	50	78	4.79	<0.0001∗∗∗	31	77	1.665	0.031∗
Female	54	72	5.404	<0.0001∗∗∗	47	79	2.104	0.0006∗∗∗

## Data Availability

The data that support the findings of this study are available from the corresponding author upon reasonable request.
